# Temporal Changes in Patient-Matched *Staphylococcus epidermidis* Isolates from Infections: towards Defining a ‘True’ Persistent Infection

**DOI:** 10.3390/microorganisms8101508

**Published:** 2020-09-30

**Authors:** Llinos G. Harris, Owen Bodger, Virginia Post, Dietrich Mack, Mario Morgenstern, Holger Rohde, T. Fintan Moriarty, Thomas S. Wilkinson

**Affiliations:** 1 Microbiology and Infectious Diseases, Institute of Life Science, Swansea University Medical School, Swansea SA2 8PP, UK; o.bodger@swansea.ac.uk (O.B.); T.S.Wilkinson@Swansea.ac.uk (T.S.W.); 2 AO Research Institute Davos, 7270 Davos, Switzerland; virginia.post@aofoundation.org (V.P.); fintan.moriarty@aofoundation.org (T.F.M.); 3 Bioscientia Labor Ingelheim, Institut für Medizinische Diagnostik GmbH, Mikrobiologie/Infektiologie, 55218 Ingelheim, Germany; dietrich.mack@bioscientia.de; 4 Department of Orthopaedic Surgery and Traumatology, University Hospital Basel, 4031 Basel, Switzerland; mario.morgenstern@usb.ch; 5 Institut für Medizinische Mikrobiologie, Virologie & Hygiene, Universitätsklinikum Hamburg-Eppendorf, Universität Hamburg, D-20246 Hamburg, Germany; rohde@uke.de

**Keywords:** *Staphylococcus epidermidis*, persistent infection, WGS, ODRI, genotype

## Abstract

*Staphylococcus epidermidis* is found naturally on the skin but is a common cause of persistent orthopaedic device-related infections (ODRIs). This study used a pan-genome and gene-by-gene approach to analyse the clonality of whole genome sequences (WGS) of 115 *S. epidermidis* isolates from 55 patients with persistent ODRIs. Analysis of the 522 gene core genome revealed that the isolates clustered into three clades, and MLST analysis showed that 83% of the isolates belonged to clonal complex 2 (CC2). Analysis also found 13 isolate pairs had different MLST types and less than 70% similarity within the genes; hence, these were defined as re-infection by a different *S. epidermidis* strain. Comparison of allelic diversity in the remaining 102 isolates (49 patients) revealed that 6 patients had microevolved infections (>7 allele differences), and only 37 patients (77 isolates) had a ‘true’ persistent infection. Analysis of the core genomes of isolate pairs from 37 patients found 110/841 genes had variations; mainly in metabolism associated genes. The accessory genome consisted of 2936 genes; with an average size of 1515 genes. To conclude, this study demonstrates the advantage of using WGS for identifying the accuracy of a persistent infection diagnosis. Hence, persistent infections can be defined as ‘true’ persistent infections if the core genome of paired isolates has ≤7 allele differences; microevolved persistent infection if the paired isolates have >7 allele differences but same MLST type; and polyclonal if they are the same species but a different MLST type.

## 1. Introduction

Persistent orthopaedic device-related infections (ODRIs) are a major clinical problem due to their chronic persistence and recalcitrance to antibiotics. The skin commensal *Staphylococcus epidermidis* is a leading cause of nosocomial ODRIs [1], as it has evolved sophisticated regulatory systems and mechanisms, allowing it to adapt to changing environmental conditions during colonization and infection [2].

Whole genome sequencing has shown that commensal and clinical *S. epidermidis* comprise highly diverse assemblages of related strains [3,4]. In addition, isolates that are closely related genetically can display different phenotypes that may allow some of them to make the transition from the skin environment to implant-associated infections [4,5]. A previous study has shown high variability in biofilm phenotypes in isolates from commensal and infection sources, and a correlation between genotypic groupings (clade, clonal complex and sequence type) and known biofilm-associated genes, thus indicating widespread acquisition and mobility of these genes between lineages [3]. It has been suggested that in-host adaptation influences the diversity within *S. aureus* [6,7] and *S. epidermidis* [4,8] from the skin and infected sites; however, there is a paucity of data on collections of persistent prosthetic joint infection (PJI) and fracture-related infection (FRI) isolates. Thus, to analyse the clonality of isolates during persistent *S. epidermidis* infections (up to 428 days), the WGS of 115 *S. epidermidis* isolates from 55 patients with persistent PJIs or FRI were examined.

## 2. Materials and Methods

### 2.1. Bacterial Isolate Collection and Genomes

Persistent infections isolates were defined as isolates from patients that required further surgery a minimum of five days after an initial infection diagnosis, with isolation of the same strain by intraoperative specimen culture [9]. The persistent infection isolate collection (Table 1 and Table S1) consisted of 92 PJI isolates (46 patients) collected between 1999 and 2004 at the ENDO-Klinik Hamburg, and University Hospital Hamburg-Eppendorf, Germany [10]; and 11 PJI isolates (4 patients) and 12 FRI isolates (5 patients), collected between 2011 and 2013 at the Trauma Center, Murnau, Germany [11,12]. The timeframe between the first isolate and subsequent isolate(s) was from 6 to 428 days. The WGS of the isolates used in this study were sequenced previously [3,12], and are archived in the Staphylococcal Bacterial Isolate Genome Sequence database (BIGSdb; https://zoo-dalmore.zoo.ox.ac.uk/bigsdb?db=staphylococcus_sheppard [13]).

### 2.2. Reference Pan-Genome

A reference pan-genome was created using the reference genomes *S. epidermidis* ATCC12228 and RP62A [14,15] and 571 *S. epidermidis* WGS available on the Staphylococcal BIGSdb database. The reference pan-genome gene list was assembled using the automated annotation pipeline RAST and the SEED database to give a functional classification and description of the predicted gene product for each gene [16]. The list was then compared to *S. epidermidis* RP62A and ATCC 12228 reference genomes, and duplicate genes were removed to create an *S. epidermidis* reference pan-genome.

### 2.3. Analysis of Persistent Infection Isolates

Using BIGSdb, the WGS of the 115 *S. epidermidis* isolates from 55 patients with a PJI or FRI (Table 1) were aligned on a gene-by-gene basis using MUSCLE software [17], and the BLAST algorithm was used to identify gene orthologs at all loci in the reference pangenome genome [3]. Variation within the population genomic sample was catalogued one gene at a time, with gene presence defined as a match with ≥70% nucleotide identity and a 50% difference in alignment length to each locus. A matrix was produced recording the presence or absence of each gene, and each new gene sequence variant was assigned a unique arbitrary allele number. For the 115 isolates, the number of shared genes and alleles (identical sequences at a given locus) were calculated and the core genome defined as the complement of genes that were present in all isolates, and the remaining genes made-up the accessory genome. The core genome was used to investigate the genetic relationship between the 115 isolates and a neighbour-joining tree implemented in MEGA6 [18] was used to estimate the genealogies for these alignments. For each pair of isolates from the same patient (the first isolate versus subsequent ones), the gene sequence data were used to determine the number of identical shared genes and allelic variants. The variability in the accessory genome was also investigated using the same method.

### 2.4. Multilocus Sequence Typing (MLST)

MLST analysis was implemented using the Thomas et al. [19] scheme in BIGSdb, and clonal complexes (CC) were determined using the eBURST algorithm [20].

### 2.5. Statistical Analyses

Data was analysed statistically using GraphPad Prism 6. The significance of the differences between the paired isolates was assessed by Generalised Liner Model (GZLM), Chi-Square or unpaired *t*-test, ANOVA, with the level of significance set at *p* ≤ 0.05.

## 3. Results

### 3.1. Descriptive Genomics for 115 Persistent Infection Isolates

**Figure 1:**
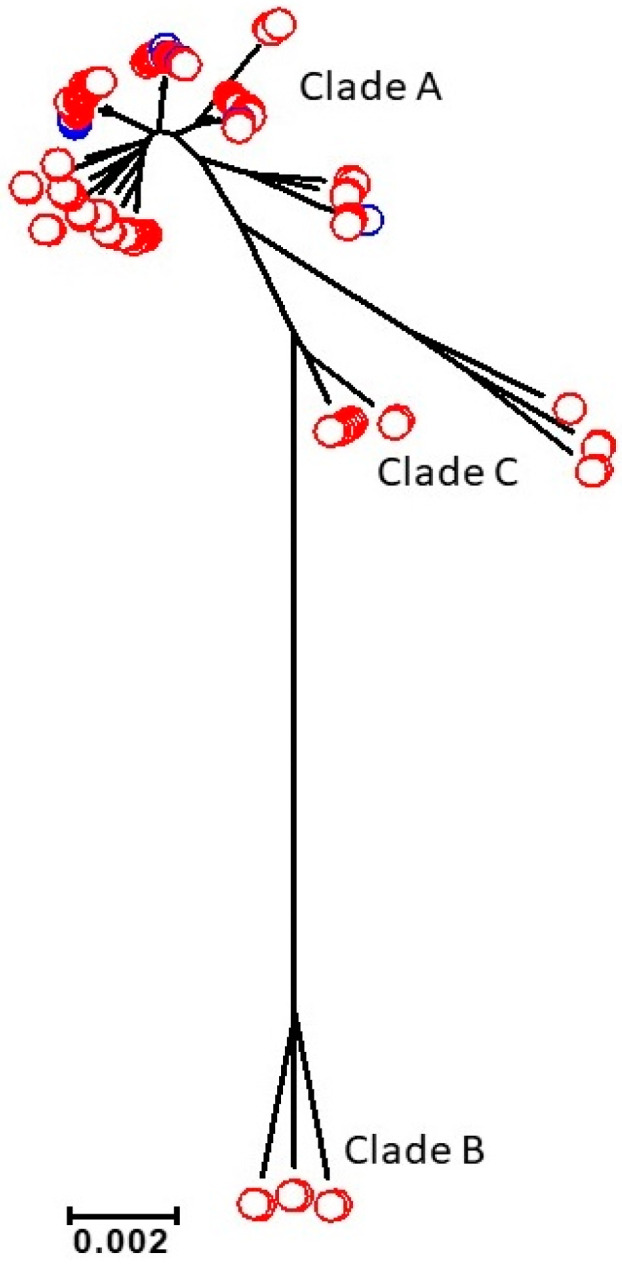
Population structure of 115 *S. epidermidis* isolates constructed from 522 core genes and implemented in MEGA6. Isolates are coloured according to source: PJI (red) and FRI (blue). The scale (0.002) is in coalescent units and represents the number of substitutions per site.

The reference pan-genome was used to investigate the genetic relationship between the 115 isolates from 55 patients with persistent infections (Table 1 and Table S1). The core genome consisted of 522 genes, and a neighbour-joining tree (Figure 1) showed that 101 isolates were found in Clade A (89%), 6 in Clade B (5%), and 8 in Clade C (7%). All the FRI isolates (*n* = 12) were found in Clade A, whilst the PJI isolates (*n* = 103) were distributed in all 3 clades (*p* = 0.33). MLST data analysis classified the isolates into 22 different sequence types (STs) confirming the genetic diversity among the isolates (Table S1). The 22 STs grouped into 1 major complex CC2 (81%), with 44% sub-clustering in CC2-I and 56% sub-clustering in CC2-II. The remaining isolates clustered either in CC23 (7%), CC66 (3%) or were singletons. Six isolates could not be assigned an MLST.

**Figure 2:**
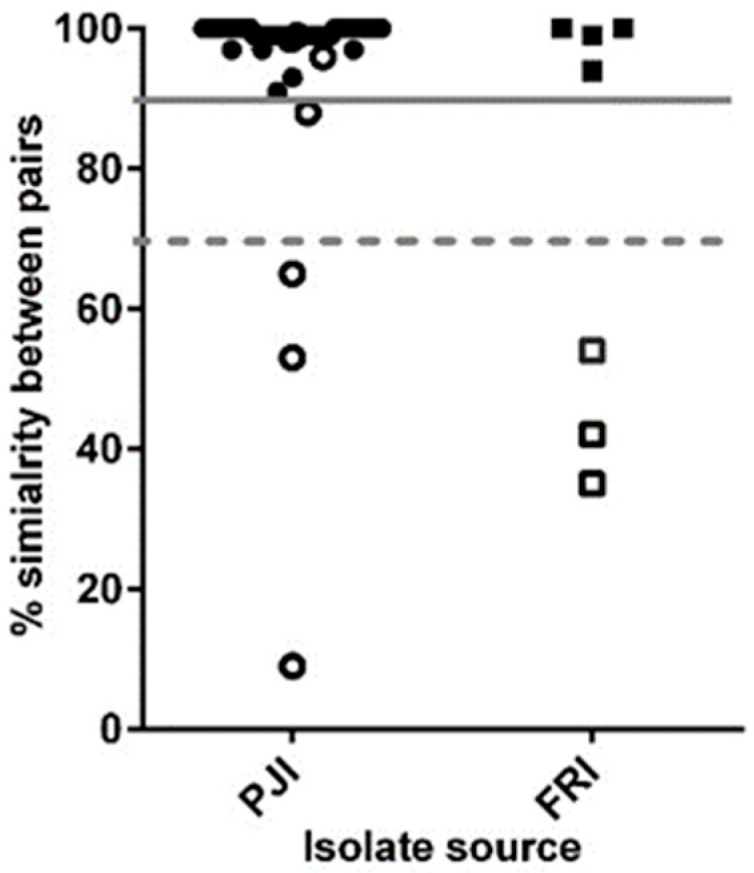
Graph showing the percentage allelic similarities in 522 core genes from 55 patients (115 isolates) based on the isolate source. Each point represents the similarity of successive isolates compared to the original isolate (paired isolates). The unbroken line indicates the 90% similarity cut-off point, hashed line the 70% similarity cut-off point, and open shapes signify patient isolate pairs with different MLST types.

The molecular diversity between the original isolate (sample 1) and each subsequent isolate from the same patient (termed paired isolates) were analysed by studying the allelic similarity (identical/non-identical) of the 522 core genes shared between paired isolates. The allelic similarity of paired isolates from 49 patients was ≥90%, but 6 patient paired isolates (12 isolates) had ≤70% allelic similarity and/or a different MLST type (open circle and squares in Figure 2), explaining the high allelic differences. Interestingly, patient P68 (PJI) had three infection episodes, where the first two isolates had a ≥90% similarity and identical MLST types but the first and third isolates had a <70% allelic similarity and different MLST types. Therefore, isolates from 6 patients (12 isolates) and third isolate from P67 were deemed to have caused an infection of the ODRI by a different strain (polyclonal [21]) rather than a persistent infection with the same strain, and were omitted from further analysis (Figure 2 and Figure S1).

### 3.2. Core Genome Analysis for 102 Persistent Infection Isolates (49 Patients)

**Figure 3:**
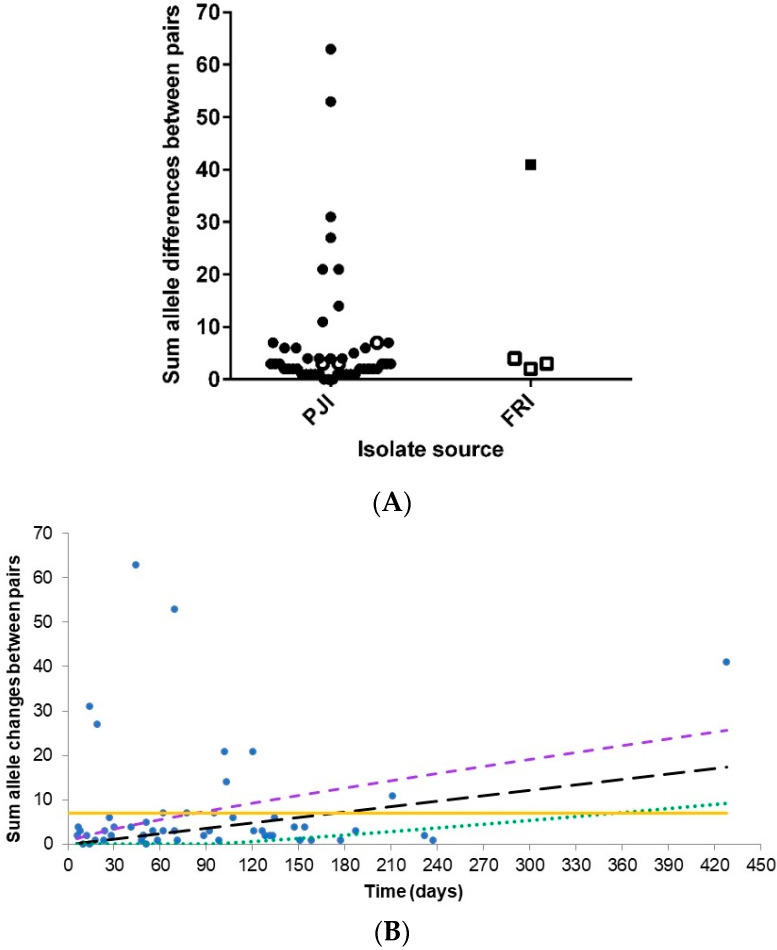
Molecular diversity among 102 isolates from 49 patients, (**A**) graph showing the sum of allele differences in 710 core genes; (**B**) frequency distribution of allele differences between observed values (blue dots) and expected values (black hash line) against time between isolate pairs as determined by Poisson distribution analysis. Purple hash line: upper limit (95% CI), green dotted line: lower limit (95% CI), and yellow line: signifies 7 allele differences.

The core genome was redefined (Figure S1) and consisted of 710 genes. The molecular diversity among the core genes of the isolate pairs was analysed again and resulted in 40 patients having fewer than 10 allele differences, 4 with 11–24 allele differences, and 5 having over 25 allele differences (Figure 3A). A GZLM was then used to analyse the frequency distribution of the allele differences between the paired isolates. The distribution of allele differences did not fit a Poisson distribution, suggesting that the rate of allele differences was not constant across the samples, even when time between isolate pairs was considered (Figure 3B). However, the results indicate that an allele difference ≤ 7 or below the upper 95% confidence limit (CI) were ‘true’ persistent infection isolates. Hence, 8 patients (16 isolates) outside these criteria were omitted from further analysis and deemed to have microevolved infections, as they share the same clonal lineage [22].

### 3.3. Analysis of the Core and Accessory Genomes of ‘True’ Persistent Infection Isolates

**Figure 4:**
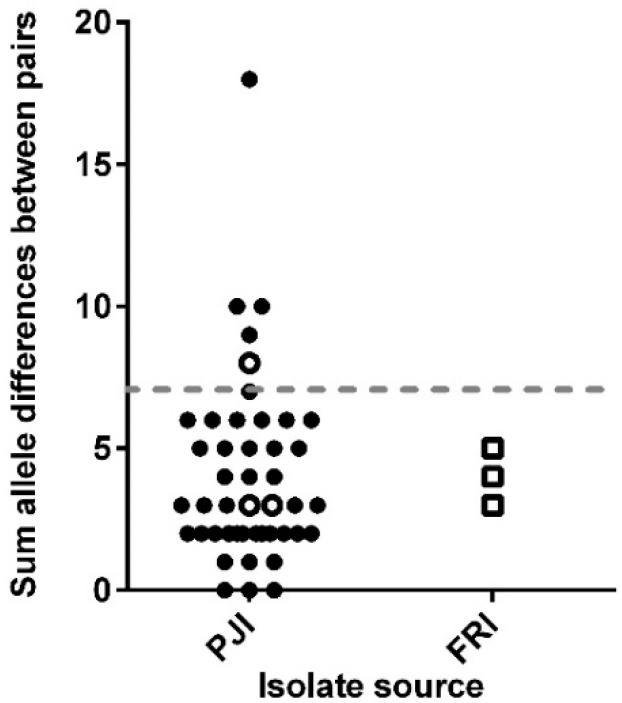
Molecular diversity among the 86 isolates from 41 patients, with the graph showing the sum of allele differences in 841 core genes. Line designates the patient pairs with ≥7 allele differences, and open symbols represent patients with more than 3 isolates.

The core genome was re-defined again based on the remaining 41 patient paired isolates (86 isolates; Figure S1) and consisted of 841 genes. The molecular diversity analysis resulted in 37 patients having ≤7 allele differences, 3 with 8–10 allele differences, and 1 having 18 allele differences (Figure 4). Further analysis found 731 of the core genes were identical between the patient paired isolates, while the remaining 110 genes had variations, with the majority of these in the paired isolates of the 4 patients with >7 allele differences but below 95% CI. The general function of the 110 genes are summarised in Table S2, with genes associated with metabolism showing the greatest differences between paired isolates. No statistical correlation was found between the sum of allele differences and the time between the paired isolates (*p* > 0.05) over the 237-day range studied here. Hence, the results suggest that 37 patients had a ‘true’ persistent infection, while the other 4 patients (9 isolates) had a microevolved persistent infection, as they shared the same clonal lineage.

**Figure 5:**
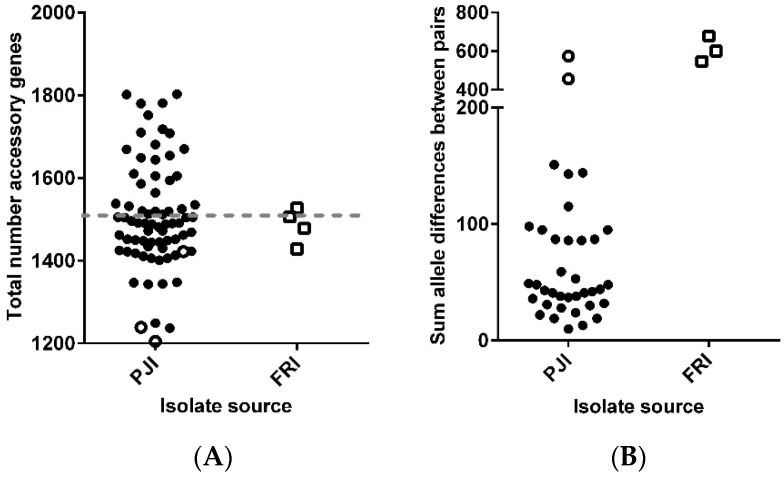
Molecular diversity within the accessory genome of the 77 isolates from 37 patients; (**A**) total number of accessory genes present (dashed line represents average number of genes); and (**B**) sum allele differences within the pairs in 2936 accessory genes. Open symbols represent patients with more than 3 isolates.

The accessory genomes of the 37 patients (77 isolates) with a ‘true’ persistent infection were examined. Analysis showed that the accessory genome consisted of 2936 genes, with an isolate having an average accessory genome of 1515 genes (Figure 5A). The sums of allele differences within the paired isolates are shown in Figure 5B, where the greatest difference was seen in P63 and P67. In 1188 (40.5%) of the accessory genes, no variation was found between paired isolates, while 1748 (59.5%) of the genes had between 1 (21.9%) and 22 (0.07%) differences. A summary of the general functions of the 1748 genes are listed in Table S3. The time between the isolation of the paired isolates had no significant effect on the sum of allele differences observed within the accessory genome (*p* < 0.05). Due to the high variation within the accessory genome, the core genome should be used to define ‘true’ persistent infections.

## 4. Discussion

ODRIs caused by the commensal bacterium *S. epidermidis* are a major clinical problem, due to their persistence and poor response to antibiotics [1]. This study used a pan-genome approach to examine the WGS of 115 *S. epidermidis* isolates from 55 patients with persistent ODRIs for clonality. The genealogical reconstruction of the 115 *S. epidermidis* isolates showed clusters distributed into three clades as previously described [3,4,23]. Consistent with clinical *S. epidermidis* isolates [4], 81% belonged to CC2, with a slight bias towards CC2-II over CC2-I [4,23].

The initial core genome analysis revealed that 6 patients (13 isolates) had polyclonal infections caused by a different *S. epidermidis* strain. The core genome of the remaining 102 isolates (49 patients) consisted of 681 genes. GZLM analysis indicated that the rate of allele differences between the paired isolates was not constant; therefore, only for those within (or close to) the expected allele difference range we can confidently say are a persistent infection and not a polyclonal or microevolved infection. Thus, a threshold of ≤7 allele differences was used to define 37 patients (77 isolates) as ‘true’ persistent infections, while 12 patients (25 isolates) had a microevolved persistent infection. This 75% incidence of persistent infection is comparable to the 71% that Galdbart et al. [24] calculated from examining the diversity in PJI caused by 54 *S. epidermidis* isolates from 14 patients using pulse-field gel electrophoresis (PFGE). Interestingly, 70 isolates from 35 patients with persistent PJIs in this study have previously been characterised by PFGE as clonally identical and not due to skin contamination [10]. However, using the WGS gene-by-gene approach to analyse allelic similarities between paired isolates, this found only 24 patients out of the 35 had a truly clonal infection.

The increased resolution of WGS allowed us to detect genetic changes during the course of persistent infections. This correlates with the findings of Van Eldere et al. [21], who also suggested that the simultaneous presence of multiple different clones in their patients was the result of genetic changes in a single infecting clone during the course of prosthetic heart valve infection, which was supported by the very close genetic relatedness observed between the four isolates. Although genetic changes occurred over time (between 6 and 428 days), it was shown that length of time between isolates had no significant influence on the sum of gene changes that occurred between paired isolates during the infection period (*p* > 0.05). Several other studies have also found that with persistent and recurrent infections relapse caused by identical or almost identical strains occurs more frequent than reinfection by a different strain [24,25,26,27].

As expected, the accessory genome in the 77 isolates (37 patients) varied significantly between the paired isolates (*p* < 0.05), despite the core genome being clonally very similar. This variability could be explained by the fact that *S. epidermidis* populations undergo frequent horizontal gene transfer and recombination resulting in a particularly diverse species [23,28]. In *S. aureus*, within-host adaptation and selective pressures have both been attributed to the mutations seen in the WGS of sequential isolates from the same person [6,7,29]. Hirschhausen et al. [30] observed that adaptive changes positively correlated with the length of *S. aureus* persistence in cystic fibrosis patients. Therefore the variability seen in this study could be the result of the patient’s own microenvironment influencing isolate variation [4,8,30,31], and it confirms that similar selective pressures are occurring and defines ‘true’ persistent infections in the related species *S. epidermidis*.

A limitation in this study and other WGS studies, is that only one isolate is collected per sample therefore they do not take into account that multiple different clones could be present in the sample, which could account for some of the variability found in particular in the microevolved infections. It has been suggested that finding a genotypically different clone during a second infection episode does not always mean reinfection but a relapse of a failed treatment of a polyclonal infection [32]. Another limitation that is part of a higher resolution analysis for the future, is that using allelic differences to assume similarity could potentially be biased as it does not consider the genetic changes (identical/non-identical) that may have occurred and what their effect would have been on the isolate phenotypes. However, in species with open pangenomes, such as *S. epidermidis* [23], single nucleotide polymorphism (SNP)-based mapping approaches are more problematic due to the diversity found in these bacteria species [33].

## 5. Conclusions

This study demonstrates how using WGS improves the accuracy of identifying persistent infection, over other techniques such as PFGE. The results showed that 18 out of the 55 patients previously reported as clonal were actually more diverse and caused by polyclonal or microevolved *S. epidermidis* strains. Therefore, the remaining 37 patients were defined as having a ‘true’ persistent infection as their paired isolates had ≤7 allele differences. The significant differences exhibited by the accessory genome demonstrates the influence within-host adaptation and selective pressures can have on *S. epidermidis* genes, something that has already been shown in *S. aureus*. Thus, the natural corollary to this being that assumptions cannot be made that *S. epidermidis* isolates collected from a persistent infection are the same causative strain as genetic changes may occur that could involve the acquisition/loss of antibiotic resistance and virulence associated genes, thereby modulating isolate pathogenicity. Such studies also have the potential to aid in the development of new treatments for persistent *S. epidermidis* infections.

## Figures and Tables

**Table 1:** Summary of the multiple patient matched infection isolates used in the study.

Isolate Source	No. Patients	No. Isolates	No. Patients with Paired Isolates	No. Patients with 3 or More Isolates	Time between Matched Paired Isolates (in Days)			
	(*n* = 55)	(*n* = 115)	(No. Isolates)	(No. Isolates)	<30	31–90	91–180	>181
Prosthetic joint infection (PJI)	50	103	48 (96)	2 (7)	13	17	20	3
Fracture-related infection (FRI) ^a^	5	12	4 (8)	1 (4)	2	0	1	4

^a^ includes isolates from infected nails (10) and thoracic internal implant (2).

## Supplementary Materials

The following are available online at https://www.mdpi.com/2076-2607/8/10/1508/s1, Figure S1: Flow diagram outlining breakdown of isolate and patient numbers at each analysis, Table S1: List of the multiple patient matched infection isolates, Table S2: Summary on the function of the 110 core genes showing variations within the 37 patient paired isolates (74 isolates), Table S3: Summary on the function of the 1976 accessory genes showing 1–21 variations between the paired isolates (74 isolates, 37 patients).
