# Supplementary material for: Temporal Changes in Patient-Matched Staphylococcus epidermidis Isolates from Infections: towards Defining a ‘True’ Persistent Infection
Source: Microorganisms. 2020 Sep 30;8(10):1508. doi: 10.3390/microorganisms8101508 (PMC7601538; doi:10.3390/microorganisms8101508)
Supplement: Supplementary file 1 [file microorganisms-08-01508-s001.pdf]

Supplemental Figure 1. Flow diagram outlining breakdown of isolate and patient numbers at each analysis.

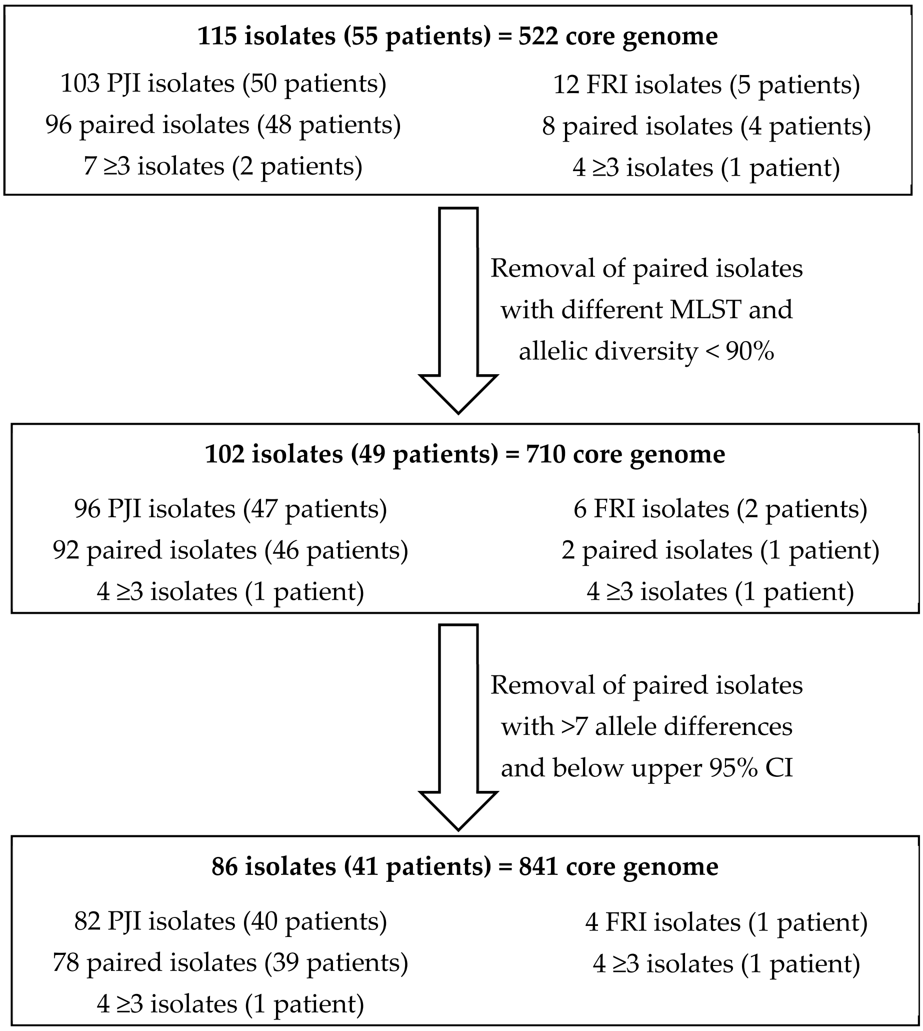

Supplemental Table 1. List of the multiple patient matched infection isolates.

Patient	Source	Time between isolates (days)			Clade	ST			
		Isolate 1&2	Isolate 1&3	Isolate 1&4		Isolate 1	Isolate 2	Isolate 3	Isolate 4
P1	PJI	14			A	171	171		
P2	PJI	62			A	2	2		
P3	PJI	41			A	2	2		
P4	PJI	18			A	59	59		
P5	PJI	121			A	2	2		
P6	PJI	51			C	51	51		
P7[#]	PJI	102			C	89	89		
P8[#]	PJI	44			A	n/a	n/a		
P9	PJI	8			B	66	66		
P10	PJI	10			B	59	59		

P11	PJI	49			A	89	89		
P12	PJI	131			A	217	217		
P13	PJI	49			C	22	22		
P14	PJI	151			C	5	5		
P15	PJI	134			A	5	5		
P16	PJI	28			A	n/a	n/a		
P17	PJI	126			A	2	2		
P18	PJI	187			A	5	5		
P19	PJI	128			A	2	2		
P20	PJI	107			A	22	22		
P21#	PJI	19			A	897	897		
P22	PJI	51			A	21	21		
P23	PJI	48			A	2	2		
P24	PJI	58			A	89	89		
P25	PJI	88			B	2	2		
P26#	PJI	69			B	88	88		
P27*	PJI	91			A	22	2		
P28	PJI	27			A	5	5		
P29	PJI	23			A	297	297		
P30	PJI	95			A	22	22		
P31	PJI	211			A	5	5		
P32	PJI	6			A	297	297		
P34	PJI	12			A	2	2		
P35	PJI	30			A	2	2		
P36	PJI	98			A	59	59		
P37	PJI	24			A	5	5		
P38	PJI	77			A	59	59		
P39#	PJI	133			A	2	2		
P40	PJI	103			A	n/a	n/a		
P41	PJI	158			A	168	168		
P42	PJI	154			A	10	10		
P43#	PJI	147			A	208	208		
P44	PJI	120			A	59	59		
P45	PJI	177			B	59	59		
P46	PJI	71			B	48	48		
P47	PJI	237			A	9	9		
P63	FRI	7	92	232	A	2	2	2	2
P64#	FRI	428			A	5	5		
P65*	FRI	119			A	48	87		
P66*	FRI	28			A	5	87		
P67	PJI	55	62	69	A	23	23	23	23
P68#	PJI	14	30		A	5	5	2*	
P69*	FRI	197			A	5	22		
P70*	FRI	257			A	57	22		
P71*	PJI	24			A	490	5		

n/a – not assigned and defined as having different MLST types.

* isolates/patients omitted after initial analysis of 117 isolates using core genome of 522 genes.

# patients omitted for having an allele difference ≤ 7 or below the 95% CI after analysis using core genome of 710 genes.

Supplemental Table 2. Summary on the function of the 110 core genes showing variations within the 37 patient paired isolates (77 isolates).

Gene function	No. genes
Metabolism	52
Protein synthesis/translation	13
Nuclease production	13
Hypothetical proteins	10
Cell wall and membrane proteins	10
Transmembrane protein	6
Metal binding	3
Antibiotic resistance	2
Transport pathway	1

Supplemental Table 3. Summary on the function of the 1748 accessory genes showing 1-22 variations between the paired isolates (77 isolates, 37 patients).

Gene function	No. genes
Hypothetical proteins	498
Metabolism	408
Nuclease production	229
Cell wall and membrane proteins	151
Transmembrane protein	104
Protein synthesis/translation	95
Transport pathway	68
Bacteriophage	52
Regulation/QS	40
Metal binding	39
Antibiotic resistance	36
Cell division	17
Toxin production	3
Unknown	2
